# Long‐Term Survival of Liver Transplantation in Alcohol‐Associated Liver Disease: Insights From National Claims Data

**DOI:** 10.1111/acer.70416

**Published:** 2026-11-09

**Authors:** Wanzhu Tu, Lauren Nephew, Yining Li, Shihui Jiang, Yang Li, Chi M. Nguyen, Jing Su, Naga Chalasani, Samer Gawrieh, Suthat Liangpunsakul

**Affiliations:** ^1^ Department of Biostatistics & Health Data Science Indiana University School of Medicine Indianapolis Indiana USA; ^2^ Division of Gastroenterology and Hepatology, Department of Medicine Indiana University School of Medicine Indianapolis Indiana USA; ^3^ Roudebush Veterans Administration Medical Center Indianapolis Indiana USA

## Abstract

**Background:**

Alcohol‐associated liver disease (ALD) is a leading indication for liver transplantation (LT) in the United States. While post‐transplant outcomes are well established, the relative survival benefit of LT compared to non‐LT remains underdefined.

**Methods:**

Using Optum's de‐identified Clinformatics Data Mart Database, we identified adults with ALD between 2017 and 2024. We used logistic regression to evaluate factors associated with LT, and Cox regression to compare survival between LT recipients and non‐recipients.

**Results:**

Among 373,025 patients with ALD, 3453 underwent LT. Female sex (adjusted OR = 0.805; 95% Confidence Interval (CI): 0.750–0.866), African‐American race (adjusted OR = 0.542; 95% CI: 0.462–0.636), older age, and non‐liver comorbidities, and all types of metastatic cancer were associated with lower odds of LT; non‐metastatic liver cancer, however, was associated with increased odds of LT (adjusted OR = 4.147; 95% CI: 3.659–4.699). The 8‐year restricted mean survival time was 2600 days (~7.12 years) for LT recipients and 1799 days (~4.93 years) for nonrecipients. The median survival among deceased patients was 1000 days with LT vs. 355 days without LT. LT was associated with a lower adjusted mortality risk (HR, 0.365; 95% CI: 0.333–0.401).

**Conclusion:**

LT confers a substantial survival advantage for patients with ALD, beyond what is achieved without transplantation. However, significant sex and race‐based disparities persist, underscoring the need for policy interventions to promote equitable access and optimized candidate selection.

## Graphical abstract

Liver transplantation was associated with substantially improved survival compared with no transplantation. Kaplan–Meier analyses demonstrated prolonged survival among transplant recipients. Among 1000 randomly selected decedents, individual survival trajectories illustrated time from index date to death, highlighting both transplantation timing and post‐transplant survival. Blue segments represent survival after transplantation, yellow segments indicate waiting time to transplantation, and red segments represent survival without transplantation. Median survival was markedly longer in transplant recipients, underscoring the survival benefit associated with liver transplantation.

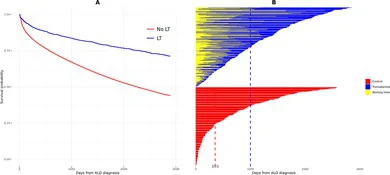

## 1 Introduction

Alcohol‐associated liver disease (ALD) has become a leading indication for liver transplantation (LT) in the United States (Lee et al. 2019), driven in part by the increase in alcohol‐related morbidity and mortality (Asrani et al. 2019; Tapper and Parikh 2018). Despite the growing burden, access to LT for patients with ALD remains inconsistent and is influenced by provider attitudes, institutional protocols, and regional variations in transplant evaluation, referral, and waitlisting practices (Herrick‐Reynolds et al. 2021; Lim et al. 2022; Marroni et al. 2018). This variability has contributed to the differing survival estimates in this population. Several studies have demonstrated the efficacy of early liver transplantation in patients with severe alcohol‐associated hepatitis (sAH). One prospective study reported a 6‐month post‐transplant survival rate of 77% among early transplant recipients compared to 23% in non‐transplant controls (Mathurin et al. 2011). A subsequent U.S.‐based cohort study found similar favorable outcomes, with a 6‐month post‐transplant survival rate of 89% versus 11% in matched controls (Im et al. 2016). In a retrospective analysis, early LT, defined as transplantation before six months of alcohol abstinence, was associated with a 1‐year survival rate of 94% in patients with sAH (Lee et al. 2018).

While most existing studies have focused on post‐transplant outcomes, initiating follow‐up at the time of liver transplantation inherently excludes the pre‐transplant period. This approach overlooks the critical window during which key clinical events, such as hepatic decompensation, progression of comorbidities, or death while awaiting transplantation, may occur. It is also within this interval that disparities in access to care and transplantation become evident. The fundamental question of how long patients with ALD live, with or without LT, remains inadequately quantified. A more comprehensive understanding of the natural history of ALD, factors associated with LT, and the extent to which LT modifies long‐term outcomes is essential to guide clinical decision‐making and inform equitable health policies.

This study aimed to provide initial estimates of short‐ and long‐term (> 3 years) survival times among patients with ALD, including both liver transplant recipients and non‐recipients, using data derived from a national medical claims database. These survival estimates serve as a foundation for quantifying the relative survival benefit associated with LT. Medical claims data are particularly valuable as they capture real‐world clinical practice. Notably, individuals covered by commercial and Medicare Advantage insurance plans constitute the largest segment of the clinical population in the United States (Keisler‐Starkey and Bunch 2020). In addition, this study offers an opportunity to assess the clinical factors associated with the likelihood of receiving LT.

## 2 Methods

We conducted a retrospective observational study using Optum Clinformatics Data Mart (CDM), a large de‐identified administrative claims database containing longitudinal medical and pharmacy claims from commercially insured and Medicare Advantage enrollees across the United States. The database provides comprehensive information on patient demographics, inpatient and outpatient diagnoses and procedures, prescription drug use, and mortality. The study period spanned from January 1, 2017, to August 31, 2024, with a 10‐year lookback window (January 1, 2007, to December 31, 2016) used to identify preexisting comorbidities. Data access was granted through Indiana University, whose Institutional Review Board classified the study as exempt due to the use of de‐identified data.

### 2.1 Organ Procurement Statement

This study used U.S. national claims data and did not involve direct organ procurement. All transplants represented in the dataset were performed in the United States under the regulatory oversight of the Organ Procurement and Transplantation Network (OPTN) and United Network for Organ Sharing (UNOS). No organs were procured from executed prisoners. All organs were procured following informed consent or authorization, in accordance with U.S. laws and regulations.

### 2.2 Study Cohort

Patients were eligible for inclusion if they had one recorded diagnosis of ALD between January 1, 2017, and August 31, 2024. ALD diagnoses were defined using ICD‐10‐CM codes (Centers for Medicare & Medicaid Services 2021), including K70.1 (alcoholic hepatitis), K70.2 (alcoholic fibrosis and sclerosis), K70.3 (alcohol‐associated cirrhosis), and K70.4 (alcoholic hepatic failure). In addition, the cohort also included individuals with diagnostic codes K74.60 and K74.69 (unspecified and other cirrhosis) if accompanied by alcohol use disorder (AUD) codes F10.1xx (alcohol abuse) and/or F10.2xx (alcohol dependence). These codes have been previously validated for the identification of ALD (Vannier et al. 2022). Prevalent cases of ALD diagnosed before January 1, 2017, were excluded. The date of the first ALD diagnosis on or after January 1, 2017, was designated as the index date for each participant and served as the starting point for follow‐up.

### 2.3 Patient Characteristics

Demographic information, including age, sex, and race/ethnicity, was extracted from Optum enrollment files. Clinical comorbidities were identified using ICD‐10 codes (Nguyen et al. 2025; Tu et al. 2024). The chronic conditions assessed included myocardial infarction, congestive heart failure, cerebrovascular disease, dementia, chronic obstructive pulmonary disease (COPD), rheumatic disease, hemiplegia, peptic ulcer disease, renal disease, cancer, and HIV/AIDS. Detailed ALD diagnoses and liver‐related complications were also recorded, including cirrhosis (alcohol‐associated and other etiologies), gastrointestinal (GI) hemorrhage, hepatorenal syndrome (HRS), renal failure, ascites, hepatic encephalopathy (HE), and hepatic failure. Additionally, individuals diagnosed with AUD, including alcohol abuse, dependence, or withdrawal, were identified and included in the analysis. The full list of relevant ICD codes is presented in Tables S1 and S2.

### 2.4 Liver Transplantation

The primary code used to identify LT procedures in the study participants was the ICD‐10‐Procedure Coding System (PCS) (Steindel 2010) code 0FY00Z0, which indicates a liver transplant from an allogeneic donor, defined as a genetically distinct donor of the same species (e.g., a cadaveric or living unrelated donor). LT was further confirmed using the Current Procedural Terminology (CPT) (Frank et al. 2022) code 47135. Individuals with the ICD code Z94.4 were excluded from the analysis as this code generally appears in outpatient records and signifies a prior history of LT rather than the transplant procedure itself. This strategy was employed to specifically capture LT procedures performed in inpatient settings.

### 2.5 Mortality

An important outcome of interest in this study was all‐cause mortality among the participants. Mortality status was ascertained from multiple sources within the Optum CDM, which is recognized for its comprehensive capture of death information (Tu et al. 2024). Optum determines mortality through deterministic linkage to several databases, including the Centers for Medicare & Medicaid Services (CMS) records, the Social Security Administration Death Master File, hospital discharge data indicating death, termination of insurance coverage due to death, linked electronic health records (EHRs), and external obituary sources. To protect patient privacy, Optum provides only the month and year of death without the exact date. Accordingly, the date of death was set to the last day of the reported month for all analyses. Although we expressed follow‐up as the number of days from the date of ALD diagnosis, the survival time had an accuracy that was only accurate to the month. Given the long follow‐up period of approximately 8 years, such a date assignment would not materially affect the overall survival estimates or conclusions.

### 2.6 Data Analysis

The demographic and clinical characteristics of study participants were stratified by LT status. Categorical variables were compared using the chi‐square test, whereas continuous variables were analyzed using *t*‐tests. A multivariable logistic regression analysis was conducted to evaluate the associations between patient characteristics and the likelihood of receiving LT. Estimated effects were reported as adjusted odds ratios (aORs) with corresponding 95% confidence intervals (CIs), after controlling for the covariates included in the model. Survival functions for LT recipients and non‐recipients were depicted using Kaplan–Meier curves, starting from the index date, the first recorded diagnosis of ALD. Participants without an event of interest were censored at the end of the observation period or the data cutoff date (August 31, 2024), whichever occurred first. To estimate the cumulative incidence of LT, death was treated as a competing risk. Restricted mean survival time (RMST) was used as a key metric to quantify survival within specific time intervals because of its intuitive interpretation as the expected survival time within a predefined window (Karrison et al. 2025; Kim et al. 2017) and its increasing preference in clinical research (Kloecker et al. 2020). RMST was calculated for LT recipients and non‐recipients over 3‐, 5‐, and 8‐year periods, and the difference in RMST between the groups was interpreted as the survival benefit conferred by LT. Finally, a Cox proportional hazards model, with LT modeled as a time‐dependent covariate, was employed to assess the effect of transplantation on survival while adjusting for patient characteristics. To examine the timing of LT following ALD diagnosis, we conducted a Cox proportional hazards regression analysis among LT recipients to identify factors associated with receiving LT within 3 years of the initial diagnosis. All statistical analyses were conducted using R software (with the tidyverse, survival, survminer, survRM2, cmprsk, tableone, broom, patchwork packages). Two‐sided *p*‐values of < 0.05 were considered statistically significant.

## 3 Results

### 3.1 Study Cohort Identification and Baseline Characteristics

The analytical cohort was derived from the Optum CDM between January 1, 2017, and August 31, 2024. A total of 42,913,798 unique individuals were enrolled in either commercial or Medicare Advantage insurance plans. Among these, 381,700 individuals had at least one ICD‐10 code indicative of ALD. After verifying the timing of the first recorded ALD diagnosis, individuals with LT dates preceding the ALD diagnosis within the study period were excluded. This resulted in a final cohort of 373,025 unique individuals, comprising 3453 LT recipients and 369,572 non‐recipients. The derivation of the study cohort is shown in Figure 1. The derived cohort served as the basis for all subsequent analyses.

**FIGURE 1:**
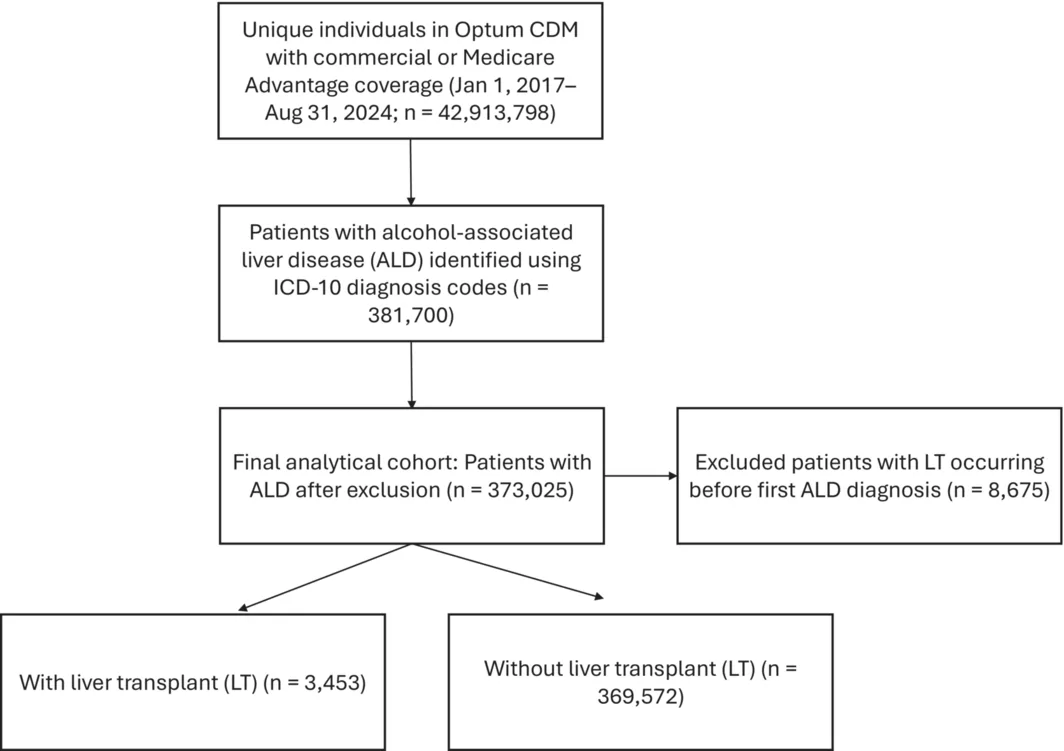
Flow diagram for the derivation of the analytical cohort from the Optum database.

The demographic and clinical characteristics of the study population are summarized in Table 1. LT recipients were significantly younger than non‐recipients (mean age 55.5 vs. 65.8 years; *p* < 0.001) and more likely to be male (62.5% vs. 55.4%; *p* < 0.001). The transplant group exhibited a lower burden of chronic comorbidities, including diabetes with complications (6.9% vs. 14.3%), congestive heart failure (3.7% vs. 16.3%), chronic obstructive pulmonary disease (6.0% vs. 18.0%), and kidney disease (9.2% vs. 15.4%). In contrast, LT recipients demonstrated a higher prevalence of alcoholic cirrhosis (22.6% vs. 14.0%), liver disease‐related complications, and signs of hepatic decompensation at diagnosis than non‐recipients, including hepatic encephalopathy (20.3% vs. 5.3%), ascites (27.4% vs. 12.9%), gastrointestinal bleeding (20.7% vs. 14.7%), and alcoholic hepatic failure (2.4% vs. 0.8%), and non‐metastatic liver cancer (9.4% vs. 2.0%). These findings indicate that LT recipients generally had more advanced and severe liver disease at the time of ALD diagnosis.

**TABLE 1:** Demographic and clinical characteristics of study participants with alcohol‐associated liver disease, by liver transplant status.

Variable	Level	No liver transplant	Liver transplant	*p*
*n*		369,572	3453	
Age (years) (mean (SD))		65.78 (12.16)	55.53 (12.35)	< 0.001
Age group (years) (%)	< 30	3273 (0.9%)	125 (3.6%)	< 0.001
	30–44	18,765 (5.1%)	426 (12.3%)	
	45–64	119,383 (32.3%)	1978 (57.3%)	
	*≥*65	228,074 (61.7%)	921 (26.7%)	
	Unknown	77 (0.0%)	3 (0.1%)	
Sex (%)	Male	204,649 (55.4%)	2159 (62.5%)	< 0.001
Race/ethnicity (%)	White	237,388 (64.2%)	2109 (61.1%)	< 0.001
	Hispanic	25,418 (6.9%)	294 (8.5%)	
	African American	36,510 (9.9%)	169 (4.9%)	
	Asian	7442 (2.0%)	87 (2.5%)	
	Unknown	62,814 (17.0)	794 (23.0)	
Diabetes (%)	No diabetes	257,978 (69.8)	2632 (76.2)	< 0.001
	Diabetes	58,814 (15.9%)	584 (16.9%)	
	Diabetes with complications	52,780 (14.3%)	237 (6.9%)	
Cancer (%)	No cancer	331,368 (89.7)	3054 (88.4)	< 0.001
	Non‐metastatic liver cancer	7439 (2.0%)	324 (9.4%)	
	Metastatic liver cancer	4777 (1.3%)	15 (0.4%)	
	Other non‐metastatic cancer	19,344 (5.2%)	52 (1.5%)	
	Other metastatic cancer	6644 (1.8%)	8 (0.2%)	
Myocardial infarction (%)	Yes	18,939 (5.1%)	31 (0.9%)	< 0.001
Congestive heart failure (%)	Yes	60,337 (16.3%)	128 (3.7%)	< 0.001
Peripheral vascular disease (%)	Yes	38,480 (10.4%)	130 (3.8%)	< 0.001
Cerebrovascular disease (%)	Yes	20,662 (5.6%)	45 (1.3%)	< 0.001
Dementia (%)	Yes	11,777 (3.2%)	5 (0.1%)	< 0.001
COPD (%)	Yes	66,421 (18.0%)	208 (6.0%)	< 0.001
Rheumatic disease (%)	Yes	8982 (2.4%)	50 (1.4%)	< 0.001
Peptic ulcer disease (%)	Yes	8785 (2.4%)	81 (2.3%)	0.949
Hemiplegia (%)	Yes	2826 (0.8%)	6 (0.2%)	< 0.001
HIV/AIDS (%)	Yes	1975 (0.5%)	15 (0.4%)	0.493
Renal disease (%)	Yes	56,843 (15.4%)	319 (9.2%)	< 0.001
Cirrhosis (%)	Yes	301,730 (81.6%)	3453 (100%)	< 0.001
GI hemorrhage (%)	Yes	54,206 (14.7%)	715 (20.7%)	< 0.001
HRS/renal failure (%)	Yes	46,805 (12.7%)	360 (10.4%)	< 0.001
Ascites (%)	Yes	47,580 (12.9%)	946 (27.4%)	< 0.001
Hepatic encephalopathy (%)	Yes	19,657 (5.3%)	702 (20.3%)	< 0.001
Alcoholic hepatic failure (%)	Yes	2978 (0.8%)	84 (2.4%)	< 0.001
Alcohol abuse, dependence or withdrawal (%)	Yes	56,031 (15.2%)	489 (14.2%)	0.108

Abbreviations: COPD, chronic obstructive pulmonary disease; GI, gastrointestinal; HIV/AIDS, human immunodeficiency/acquired immunodeficiency syndrome; HRS, hepatorenal syndrome.

### 3.2 Factors Associated With LT Receipt

The estimated associations between patient characteristics and LT recept are summarized in Table 2. Multivariate logistic regression analysis demonstrated that female sex (adjusted odds ratio [aOR] = 0.805; 95% confidence interval [CI]: 0.750–0.866; *p* < 0.001) and older age (aOR = 0.948; 95% CI: 0.946–0.950; *p* < 0.001) were significantly associated with reduced odds of receiving LT. Compared to White individuals, African Americans had lower odds of receiving LT (aOR = 0.542; 95% CI: 0.462–0.636; *p* < 0.001).

**TABLE 2:** Factors associated with receipt of liver transplantation in multivariable logistic regression. Effects are expressed as adjusted odds ratios and 95% confidence limits.

Variable	Adjusted odds ratio	Lower 95% confidence limit	Upper 95% confidence limit	*p*
(Intercept)	0.128	0.108	0.151	< 0.001
Female	0.805	0.750	0.866	< 0.001
Age	0.948	0.946	0.950	< 0.001
*Race/ethnicity (ref: White)*				
Hispanic ethnicity	1.112	0.981	1.261	0.098
African American race	0.542	0.462	0.636	< 0.001
Asian race	1.024	0.820	1.279	0.836
Unknown race	0.928	0.851	1.012	0.090
*Diabetes (ref: No diabetes)*				
Diabetes	1.013	0.923	1.112	0.783
Diabetes with complications	0.671	0.580	0.778	< 0.001
*Cancer (ref: No cancer)*				
Non‐metastatic liver cancer	4.147	3.659	4.699	< 0.001
Metastatic liver cancer	0.199	0.119	0.334	< 0.001
Other non‐metastatic cancer	0.380	0.288	0.502	< 0.001
Other metastatic cancer	0.132	0.066	0.265	< 0.001
Myocardial infarction	0.363	0.253	0.522	< 0.001
Congestive heart failure	0.331	0.274	0.400	< 0.001
Peripheral vascular disease	0.664	0.553	0.796	< 0.001
Cerebral vascular disease	0.430	0.318	0.581	< 0.001
Dementia	0.120	0.050	0.289	< 0.001
COPD	0.426	0.369	0.492	< 0.001
Rheumatoid disease	0.728	0.547	0.969	0.029
Peptic ulcer disease	0.803	0.632	1.020	0.072
Hemiplegia	0.393	0.173	0.890	0.025
HIV/AIDS	0.565	0.337	0.948	0.031
Renal disease	1.105	0.965	1.266	0.150
Alcoholic‐associated cirrhosis	2.267	2.048	2.509	< 0.001
Other & unspecified cirrhosis	2.728	2.478	3.003	< 0.001
GI hemorrhage	1.219	1.112	1.336	< 0.001
Hepatorenal syndrome	0.950	0.834	1.082	0.438
Ascites	2.367	2.173	2.578	< 0.001
Hepatic Encephalopathy	3.391	3.074	3.740	< 0.001
Alcoholic Hepatic Failure	1.460	1.141	1.867	0.003
Alcohol use disorder	0.459	0.410	0.514	< 0.001

Abbreviations: COPD, chronic obstructive pulmonary disease; GI, gastrointestinal; HIV/AIDS, human immunodeficiency/acquired immunodeficiency syndrome; HRS, hepatorenal syndrome.

Among chronic comorbidities, the presence of diabetes with complications (aOR = 0.671; 95% CI, 0.580–0.778; *p* < 0.001), congestive heart failure (aOR = 0.331; 95% CI: 0.274–0.400; *p* < 0.001), myocardial infarction (aOR = 0.363; 95% CI: 0.253–0.522; *p* < 0.001), chronic obstructive pulmonary disease (COPD, aOR = 0.426; 95% CI, 0.369–0.492; *p* < 0.001), cerebrovascular disease (aOR = 0.430; 95% CI: 0.318–0.581; *p* < 0.001), and dementia (aOR = 0.120; 95% CI: 0.050–0.289; *p* < 0.001) were associated with significantly lower adjusted odds of LT after controlling in the multivariable model.

Conversely, diagnoses reflecting advanced liver disease were strongly associated with an increased likelihood of LT. Individuals with non‐metastatic liver cancer (aOR = 4.147; 95% CI: 3.659–4.699; *p* < 0.001) and individuals with alcohol‐associated cirrhosis (aOR = 2.267; 95% CI: 2.048–2.509; *p* < 0.001) had more than quadrupled and double the odds of undergoing LT, respectively. Markers of decompensated liver disease, including ascites (aOR = 2.367; 95% CI: 2.173–2.578; *p* < 0.001), GI bleeding (aOR = 1.219; 95% CI: 1.112–1.336; *p* < 0.001), and HE (aOR = 3.391; 95% CI: 3.074–3.740; *p* < 0.001), were also significantly associated with higher odds of LT. Notably, patients diagnosed with AUD (including alcohol abuse, dependence, or withdrawal) exhibited significantly reduced odds of transplantation (aOR = 0.459; 95% CI: 0.410–0.514; *p* < 0.001).

### 3.3 Survival Benefit of LT in ALD

Figure 2 (panels A and B) visually demonstrates the survival advantage conferred by LT in patients with ALD. The primary outcome was all‐cause mortality. In Figure 2A, Kaplan–Meier survival curves demonstrate that LT recipients (blue curve) had significantly higher survival probabilities over time than non‐recipients (red curve). The divergence of the curves occurred early and progressively widened, indicating a substantial and sustained survival benefit associated with LT. From these Kaplan–Meier estimates, RMST was calculated for LT recipients and non‐recipients over the 3‐, 5‐, and 8‐year follow‐up periods. The RMST quantifies the expected average survival time within a specified time horizon. As shown in Table 3, LT recipients had significantly higher RMST than non‐recipients across all evaluated time windows, with significant between‐group differences. For example, within the 8‐year RMST window, evaluated over up to 2890 days, LT recipients had an RMST of 2600 days compared with 1799 days for non‐recipients, corresponding to an absolute survival gain of 801 days and a 45% longer restricted mean survival time. Figure 2B further illustrates the survival times among patients who died during the observation period, stratified by LT status. Each horizontal bar represents an individual patient, with red bars indicating non‐transplanted patients, blue bars indicating transplanted patients, and yellow segments representing pre‐transplant waiting time, that is, the time from ALD diagnosis to LT. The median survival among deceased non‐transplant patients was notably shorter (355 days) than that of LT recipients, whose median post‐diagnosis survival exceeded 1000 days. Collectively, these findings highlight a significant reduction in mortality associated with LT in patients with ALD.

**FIGURE 2:**
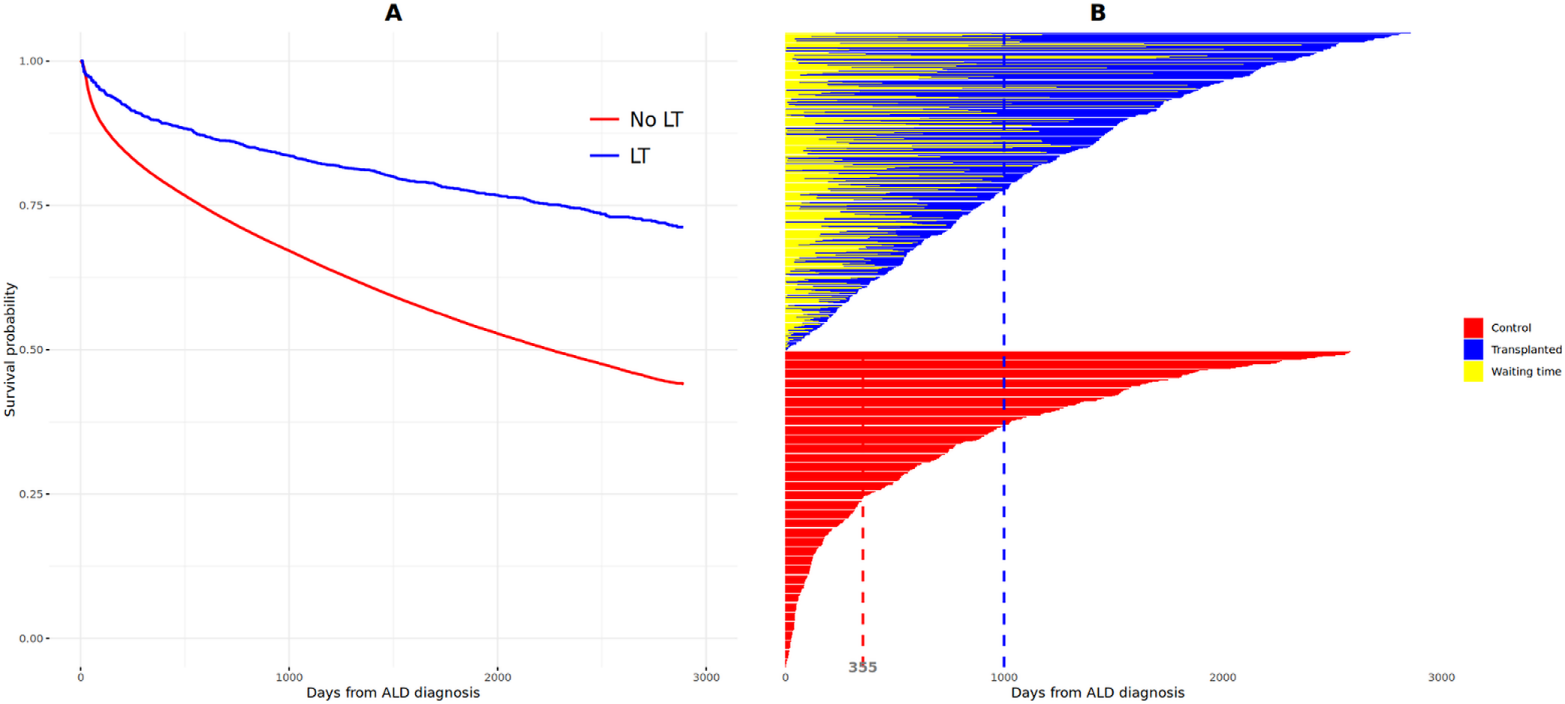
(A) Kaplan–Meier estimates of survival functions in those who received liver transplants versus those who did not receive liver transplants. (B) Histograms of the observed times to death among 1000 randomly selected individuals who died during the study observational window. Each horizontal line represents the length of survival from the index date. Blue lines indicate those who received a liver transplant, while yellow segments indicate the time they waited for transplantation. Red lines indicate times to death among individuals who did not receive a liver transplant. Vertical dash lines marked the observed median survival times.

**TABLE 3:** Restricted Mean Survival Times (RMSTs) and the associated 95% confidence intervals (CIs) among liver transplant recipients and non‐recipients.

Time window	LT, days (95% CI)	No LT, days (95% CI)	Difference, days (95% CI)	*p*	Ratio (95% CI)	*p*
3‐year	1053 (1047, 1058)	842 (840, 843)	211 (205, 217)	< 0.001	1.25 (1.24, 1.26)	< 0.001
5‐year	1708 (1695, 1720)	1278 (1276, 1281)	430 (417, 442)	< 0.001	1.34 (1.33, 1.35)	< 0.001
8‐year^a^	2600 (2574, 2625)	1799 (1795, 1803)	801 (775, 826)	< 0.001	1.45 (1.43, 1.46)	< 0.001

*Note:* RMST differences represent the additional restricted mean survival time (in days) for liver transplant (LT) recipients and non‐recipients within the prespecified time windows (3, 5, and 8 years). RMST ratios represent the relative RMST for the LT recipients compared with non‐recipients.

^a^ The 8‐year RMST window was evaluated up to 2890 days, the maximum common follow‐up time in both groups.

### 3.4 Multivariable Cox Proportional Hazards Analysis of Mortality Risk in ALD Patients

To quantify the effect of LT on all‐cause mortality while adjusting for the effects of patient characteristics, we conducted a Cox proportional hazards regression analysis by treating LT as a time‐dependent covariate. The results, summarized in Table 4, showed that LT was associated with a significantly reduced risk of death (hazard ratio [HR] = 0.365; 95% CI: 0.333–0.401; *p* < 0.001), indicating an approximately 63% reduction in the mortality hazard compared to patients who did not undergo LT.

**TABLE 4:** The estimated hazard ratios (95% confidence limits) for patient characteristics associated with mortality from the Cox proportional hazards model; liver transplant was treated as a time‐dependent variable.

Variable	Adjusted hazard ratio (HR)	Lower 95 confidence limit	Upper 95% confidence limit
Liver transplant	0.3651	0.3328	0.4006
Female	0.8908	0.8811	0.9005
Age	1.0390	1.0385	1.0396
*Race/ethnicity (ref: White)*			
Hispanic ethnicity	0.9432	0.9236	0.9631
African American race	0.9572	0.9405	0.9742
Asian race	0.6906	0.6624	0.7201
Unknown race	0.9621	0.9474	0.9770
*Diabetes (ref: No diabetes)*			
Diabetes	1.0286	1.0139	1.0435
Diabetes with complications	1.0900	1.0738	1.1064
*Cancer (ref: No cancer)*			
Non‐metastatic liver cancer	2.3264	2.2621	2.3925
Metastatic liver cancer	5.1333	4.9747	5.2969
Other non‐metastatic cancer	1.3261	1.2996	1.3532
Other metastatic cancer	3.0329	2.9499	3.1183
Myocardial infarction	1.1115	1.0896	1.1338
Congestive heart failure	1.4169	1.3972	1.4370
Peripheral vascular disease	1.0664	1.0505	1.0827
Cerebral vascular disease	1.1579	1.1355	1.1807
Dementia	1.4146	1.3828	1.4471
COPD	1.2134	1.1981	1.2288
Rheumatoid disease	1.0289	0.9969	1.0619
Peptic ulcer disease	0.9900	0.9596	1.0214
Hemiplegia	1.3377	1.2734	1.4053
HIV/AIDS	1.0301	0.9567	1.1091
Renal disease	1.1865	1.1687	1.2046
Alcoholic‐associated cirrhosis	1.0888	1.0691	1.1089
Other & unspecified cirrhosis	0.8352	0.8212	0.8495
GI hemorrhage	1.1060	1.0901	1.1222
Hepatorenal syndrome	1.5359	1.5131	1.5590
Ascites	2.0796	2.0519	2.1075
Hepatic Encephalopathy	1.7085	1.6766	1.7410
Alcoholic Hepatic Failure	1.4640	1.3946	1.5368
Alcohol use disorder	0.9752	0.9592	0.9915

Abbreviations: COPD, chronic obstructive pulmonary disease; GI, gastrointestinal; HIV/AIDS, human immunodeficiency/acquired immunodeficiency syndrome; HRS, hepatorenal syndrome.

As expected, several liver‐related conditions and complications were strongly associated with an increased mortality risk. Among patients with liver decompensation or liver disease complications, the highest hazard was observed for ascites (HR = 2.080; 95% CI: 2.052–2.108; *p* < 0.001), HE (HR = 1.709; 95% CI: 1.677–1.741; *p* < 0.001), hepatorenal syndrome (HR = 1.536; 95% CI: 1.513–1.559; *p* < 0.001), alcoholic hepatic failure (HR = 1.464; 95% CI: 1.395–1.537; *p* < 0.001), and GI bleeding (HR = 1.106; 95% CI: 1.090–1.122; *p* < 0.001).

Several other comorbid conditions were also associated with an elevated mortality risk. Among cancer‐related comorbidities, metastatic liver cancer was associated with the highest mortality risk (HR = 5.133; 95% CI: 4.975–5.297; *p* < 0.001), followed by other metastatic cancer (HR = 3.033; 95% CI: 2.950–3.118; *p* < 0.001), non‐metastatic liver cancer (HR = 2.326; 95% CI: 2.262–2.393; *p* < 0.001), and other non‐metastatic cancer (HR = 1.326; 95% CI: 1.300–1.353; *p* < 0.001). Among non‐cancer comorbidities, dementia was associated with increased mortality risk (HR = 1.415; 95% CI: 1.383–1.447; *p* < 0.001), followed by congestive heart failure (HR = 1.417; 95% CI: 1.397–1.437; *p* < 0.001), renal disease (HR = 1.187; 95% CI: 1.169–1.205; *p* < 0.001), and COPD (HR = 1.213; 95% CI: 1.198–1.229; *p* < 0.001). Additional factors associated with an elevated mortality risk included cerebrovascular disease (HR = 1.158; 95% CI: 1.136–1.181; *p* < 0.001) and hemiplegia (HR = 1.338; 95% CI: 1.273–1.405; *p* < 0.001). Alcohol‐associated cirrhosis (HR = 1.089; 95% CI: 1.069–1.109; *p* < 0.001) remained a significant risk factor for mortality. Among the demographic characteristics, female sex (HR = 0.891; 95% CI, 0.881–0.901; *p* < 0.001) and African American race (HR = 0.957; 95% CI: 0.941–0.974; *p* < 0.001) were independently associated with a reduced mortality risk, whereas older age was associated with an elevated risk (HR = 1.039 per year; 95% CI: 1.039–1.040 per year; *p* < 0.001).

A separate analysis showed that among LT recipients within 3 years of ALD diagnosis, hepatorenal syndrome, hepatic encephalopathy, ascites, and non‐metastatic liver cancer were associated with an increased chance of receiving LT. See Table S3.

## 4 Discussion

LT is an established treatment for patients with advanced ALD. Clinical guidelines from major gastroenterology and hepatology societies (Crabb et al. 2020; Jophlin et al. 2024; Thursz et al. 2018) all endorse LT for patients with advanced ALD who meet the specific selection criteria. In clinical practice, LT is increasingly being utilized as a life‐saving intervention for individuals who do not respond to other therapies. ALD is currently the most common indication for LT among young American adults (Philip et al. 2022). These guideline recommendations are primarily based on post‐transplant survival data derived from observational studies involving carefully selected patients (Im et al. 2016; Mathurin et al. 2011). Evidence regarding short‐ and medium‐term post‐LT survival is generally robust and consistent; however, data on long‐term survival (≥ 3 years) remain limited, with the notable exception of a national study that utilized the United Network for Organ Sharing database (Lee et al. 2019). Most prior investigations have focused on the post‐transplant survival benefit of LT. While this is important, examining post‐transplant survival alone provides an incomplete picture of the overall clinical trajectory following an ALD diagnosis. Anchoring survival analyses at the time of the initial ALD diagnosis allows for a more comprehensive characterization of disease progression, including the long‐term survival impact of LT. Therefore, quantifying survival time by LT status offers a valuable opportunity to assess the comparative survival advantage of LT versus no LT in patients with ALD.

To this end, this study provides important complementary information to previously published post‐transplant survival data, offering a novel perspective on the impact of LT on patient outcomes. The Kaplan–Meier survival estimates in Figure 2A demonstrate an early and sustained divergence between patients who underwent LT and those who did not, with LT recipients consistently exhibiting higher survival probabilities over the 8‐year follow‐up period. This finding is particularly noteworthy given that LT recipients are generally presumed to have more severe disease and poorer prognosis, yet they demonstrate longer average survival. Although causality cannot be definitively established given the observational study design, the survival advantage associated with LT remained statistically significant in the Cox proportional hazards model after adjustment for demographic and clinical covariates. The observed 63% reduction in the mortality hazard associated with LT corroborates the substantial survival benefit indicated by the unadjusted analysis. A key component of this research is the quantification of the survival advantage conferred by LT. RMST is a particularly relevant metric in this context, as it reflects the expected survival duration within a predefined time interval. Using RMST estimates at 3‐, 5‐, and 8‐year intervals, we demonstrated that LT recipients experienced additional survival gains of 211, 430, and 801 days, respectively, compared to non‐recipients. Correspondingly, the RMST ratios indicated that patients who underwent LT lived 25%–45% longer, on average, over these timeframes. These differences are clinically significant and highlight the sustained survival benefits associated with LT, extending beyond short‐term outcomes. Importantly, RMST offers a more intuitive and robust measure of the LT effect than hazard ratios, particularly in the setting of non‐randomized observational data.

Survival duration differed markedly between LT recipients and non‐recipients among the patients who died during the study period. As illustrated in Figure 2B, individuals who underwent LT survived nearly three times longer before death, indicating that transplantation substantially prolonged life, even among those with ultimately fatal outcomes. Since the analysis considered all‐cause mortality rather than liver‐specific death, these findings highlight the broader survival benefit conferred by LT irrespective of the cause of death.

A distinctive feature of this study was the comprehensive evaluation of factors influencing both LT access and all‐cause mortality. Our findings revealed clear disparities in LT access. Women and African‐American patients were less likely to receive LT than men and white patients, whereas Hispanic individuals had higher odds of transplantation. These disparities persisted even after adjusting for comorbidities and liver disease severity, suggesting that structural inequities rather than clinical appropriateness may underlie these differences. This observation is consistent with previous studies reporting racial and sex‐based disparities in transplant access despite standardized evaluation protocols (Mathur et al. 2010; Volk et al. 2009). Notably, these demographic groups did not demonstrate proportionally worse survival outcomes, reinforcing the notion that clinical factors alone do not fully explain disparities in LT allocation.

Patient characteristics exert complex and sometimes competing influences on the likelihood of LT and subsequent outcomes. Here, non‐metastatic liver cancer is associated with more than quadrupled odds of LT in comparison to individuals without cancer diagnoses, a finding that is consistent with an earlier report (Ishaque et al. 2019). Similarly, indicators of liver decompensation, including ascites, HE, and GI bleeding, as well as the diagnosis of alcoholic cirrhosis, were associated with significantly increased odds of receiving LT, possibly reflecting the clinical prioritization of patients with advanced disease in transplantation decision‐making. However, these same clinical features were independently associated with poorer outcomes, suggesting that their influence on patient prognosis extends beyond what LT alone can mitigate. The timing of transplantation may have been suboptimal for some patients, as prior research indicates that delayed referral or advanced disease at evaluation negatively affects transplant eligibility and survival (Goldberg et al. 2012). Transplant decisions and ALD outcomes occur in the broader context of comorbid conditions. It is therefore not surprising that chronic non‐liver‐related comorbidities, such as congestive heart failure, COPD, cerebrovascular disease, and dementia, were associated with a significantly reduced likelihood of receiving LT but also correlated with increased all‐cause mortality. These findings reflect the need for appropriate clinical caution in transplant eligibility assessments. Similarly, patients with documented AUD were less than half as likely to undergo LT, which is consistent with the current clinical practice. As anticipated, the presence of AUD, which underlies the majority of ALD cases, is associated with an increased mortality risk. This underscores the inherent tension in transplant candidacy decisions, where concerns regarding relapse and adherence may outweigh clinical indications despite evidence that carefully selected patients with AUD achieve post‐transplant outcomes comparable to those with other etiologies (Im et al. 2016; Mathurin et al. 2011). Collectively, these results illustrate the complex interplay among liver disease severity, comorbidity burden, alcohol use, and systemic inequities in influencing transplant allocation and patient outcomes. Moreover, they highlight the value of real‐world data in elucidating how these factors manifest outside of controlled clinical trials and designed cohort studies.

This study had several notable strengths. First, it leverages a large, nationally representative administrative claims database encompassing both commercial and Medicare Advantage populations, thereby reflecting real‐world clinical practices across diverse geographic regions in the United States. This broad coverage enhances the generalizability of the findings and provides a comprehensive perspective on the natural history and outcomes of ALD patients, including those who do and do not receive LT. The use of a 10‐year lookback window allows for the robust identification of pre‐existing comorbidities, improving adjustment for potential confounders in survival analyses. Additionally, by incorporating transplantation as a time‐dependent covariate and applying RMST metrics, this study offers a nuanced and clinically interpretable quantification of the survival benefits associated with liver transplantation. However, this study has several limitations that must be acknowledged. The reliance on administrative claims data, while advantageous for scale and scope, inherently carries risks of misclassification or coding inaccuracies, especially when differentiating among liver disease etiologies and capturing clinical details, such as the severity of alcohol use or adherence to abstinence. The absence of granular clinical variables, including laboratory parameters, MELD and Child‐Pugh scores, and psychosocial assessments, restricts the ability to fully characterize disease severity and transplant candidacy. In addition, MELD 3.0 was implemented in July 2023 to improve equity in liver allocation. Because long‐term follow‐up after its implementation is not yet available in the current data, we were unable to formally evaluate whether transplant access or survival outcomes changed after MELD 3.0. Furthermore, mortality data in the database are limited to months and years, thus reducing the precision of survival time estimates. Finally, the study population included only insured individuals, potentially limiting its applicability to uninsured or Medicaid populations, where barriers to transplantation may differ substantially. Future studies should compare these findings with those from patients with other liver disease etiologies to determine whether the observed patterns in LT receipt and survival are specific to ALD or reflect broader barriers to liver transplantation access and outcomes.

In summary, our study provides compelling evidence for the significant survival benefit conferred by LT in patients with ALD. Despite the severity of illness at diagnosis among transplant recipients, LT was associated with a 30% increase in expected survival time and > 60% reduction in mortality hazard after adjusting for confounders. These findings highlight the critical role of transplantation in improving long‐term outcomes of patients with advanced ALD. However, disparities in access to LT across demographic and clinical groups highlight the need for ongoing efforts to ensure equitable referrals and evaluations. Future research integrating richer clinical data and patient‐centered outcomes will be essential to optimize selection criteria and post‐transplant management, ultimately enhancing care for this vulnerable population.

## Author Contributions

Conceptualization: Wanzhu Tu, Samer Gawrieh, and Suthat Liangpunsakul. Data collection and preparation: Shihui Jiang, Yining Li, Chi M. Nguyen, Jing Su. Statistical methodology and data analysis: Yang Li, Yining Li, Shihui Jiang, and Wanzhu Tu. Visualization: Yining Li and Shihui Jiang, Yang Li, Jing Su. Data interpretation: Wanzhu Tu, Samer Gawrieh, Suthat Liangpunsakul, Lauren Nephew, Naga Chalasani. Writing: Wanzhu Tu. Critical revision: Wanzhu Tu, Samer Gawrieh, Suthat Liangpunsakul, Naga Chalasani. Funding acquisition: Wanzhu Tu and Samer Gawrieh.

## Funding

This work was supported by the National Institute on Alcohol Abuse and Alcoholism (U24AA026969).

## Conflicts of Interest

The authors declare that they have no affiliations with or involvement in any organization or entity with any financial interest in the subject matter or materials discussed in this manuscript.

## Supporting information


**Table S1:** List of ICD‐9 and 10 codes used to identify major comorbidities.
**Table S2:** ICD‐10 Codes used to identify liver‐related conditions and liver decompensation.
**Table S3:** Factors associated with time to liver transplantation within 3 years among patients who received liver transplantation.

## Data Availability

The data that support the findings of this study are available from the corresponding author upon reasonable request.
