# Supplementary material for: Long‐Term Survival of Liver Transplantation in Alcohol‐Associated Liver Disease: Insights From National Claims Data
Source: Alcohol Clin Exp Res (Hoboken). 2026 Nov 9;50(11):e70416. doi: 10.1111/acer.70416 (PMC13746985; doi:10.1111/acer.70416)
Supplement: Supplementary file 1 [file ACER-50-0-s001.docx]

**SUPPLEMENTARY MATERIALS**

**Long-term Survival of Liver Transplantation in Alcohol-associated Liver Disease:**

**Insights from National Claims Data**

Wanzhu Tu ^1#^, Lauren Nephew ^2^, Yining Li ^1^, Shihui Jiang ^1^, Yang Li ^1^, Chi M Nguyen ^1^, Jing Su ^1^,

Naga Chalasani ^2^, Samer Gawrieh ^2^, Suthat Liangpunsakul ^2,3#^

**Running title**: Liver Transplantation in Alcohol-associated Liver Disease

^1^ Department of Biostatistics & Health Data Science,

^2^ Division of Gastroenterology and Hepatology, Department of Medicine,

Indiana University School of Medicine, Indianapolis, IN 46202

^3^ Roudebush Veterans Administration Medical Center, Indianapolis, IN 46202

**Supplementary Table S1:** List of ICD-9 and 10 codes used to identify major comorbidities

| Comorbidity Condition | ICD-9 Code(s) | ICD-10 Code(s) |
| --- | --- | --- |
| Myocardial Infarction | 410, 412 | I21, I22, I252 |
| Congestive Heart Failure | 39891, 40201, 40211, 40291, 40401, 40403, 40411, 40413, 40491, 40493, 4254, 4255, 4256, 4257, 4258, 4259, 428 | I099, I110, I130, I132, I255, I420, I425, I426, I427, I428, I429, I43, I50, P290 |
| Peripheral Vascular Disease | 0930, 4373, 440, 441, 4431, 4432, 4433, 4434, 4435, 4436, 4437, 4438, 4439, 5571, 5579, V434 | I70, I71, I731, I738, I739, I771, I790, I792, K551, K558, K559, Z958, Z959 |
| Cerebrovascular Disease | 36234, 430, 431, 432, 433, 434, 435, 436, 437, 438 | G45, G46, H340, I60, I61, I62, I63, I64, I65, I66, I67, I68, I69 |
| Chronic Pulmonary Disease | 4168, 4169, 490, 491, 492, 493, 494, 495, 496, 497, 498, 499, 500, 501, 502, 503, 504, 505, 5064, 5081, 5088 | I278, I279, J40, J41, J42, J43, J44, J45, J46, J47, J60, J61, J62, J63, J64, J65, J66, J67, J684, J701, J703 |
| Rheumatic Disease | 4465, 7100, 7101, 7102, 7103, 7104, 7140, 7141, 7142, 7148, 725 | M05, M06, M315, M32, M33, M34, M351, M353, M360 |
| Peptic Ulcer Disease | 531, 532, 533, 534 | K25, K26, K27, K28 |
| Liver Disease, Mild | 07022, 07023, 07032, 07033, 07044, 07054, 0706, 0709, 570, 571, 5733, 5734, 5738, 5739, V427 | B18, K700, K701, K702, K703, K709, K713, K714, K715, K717, K73, K74, K760, K762, K763, K764, K768, K769, Z944 |
| Liver Disease, Moderate to Severe | 4560, 4561, 4562, 5722, 5723, 5724, 5728 | I850, I859, I982, I864, K704, K711, K721, K729, K765, K766, K767 |
| Diabetes without Complications | 2500, 2501, 2502, 2503, 2508, 2509 | E100, E101, E106, E108, E109, E110, E111, E116, E118, E119, E130, E131, E136, E138, E139 |
| Diabetes with Complications | 2504, 2505, 2506, 2507 | E102, E103, E104, E105, E107, E112, E113, E114, E115, E117, E132, E133, E134, E135, E137 |
| Dementia | 290, 2941, 3312 | F00, F01, F02, F03, F051, G30, G311 |
| Renal Disease | 40301, 40311, 40391, 40402, 40403, 40412, 40413, 40492, 40493, 582, 5830, 5831, 5832, 5833, 5834, 5835, 5836, 5837, 585, 586, 5880, V420, V451, V56 | I120, I131, N032, N033, N034, N035, N036, N037, N052, N053, N054, N055, N056, N057, N18, N19, N250, Z490, Z491, Z492, Z940, Z992 |
| Hemiplegia or Paraplegia | 3341, 342, 343, 3440, 3441, 3442, 3443, 3444, 3445, 3446, 3449 | G041, G114, G801, G802, G81, G82, G830, G831, G832, G833, G834, G839 |
| Cancer | 140, 141, 142, 143, 144, 145, 146, 147, 148, 149, 150, 151, 152, 153, 154, 155, 156, 157, 158, 159, 160, 161, 162, 163, 164, 165, 166, 167, 168, 169, 170, 171, 172, 174, 175, 176, 177, 178, 179, 180, 181, 182, 183, 184, 185, 186, 187, 188, 189, 190, 191, 192, 193, 194, 195, 200, 201, 202, 203, 204, 205, 206, 207, 208, 2386 | C00, C01, C02, C03, C04, C05, C06, C07, C08, C09, C10, C11, C12, C13, C14, C15, C16, C17, C18, C19, C20, C21, C22, C23, C24, C25, C26, C30, C31, C32, C33, C34, C37, C38, C39, C40, C41, C43, C45, C46, C47, C48, C49, C50, C51, C52, C53, C54, C55, C56, C57, C58, C60, C61, C62, C63, C64, C65, C66, C67, C68, C69, C70, C71, C72, C73, C74, C75, C76, C81, C82, C83, C84, C85, C88, C90, C91, C92, C93, C94, C95, C96, C97 |
| Metastatic Solid Tumour | 196, 197, 198, 199 | C77, C78, C79, C80 |
| AIDS/HIV | 042, 043, 044 | B20, B21, B22, B24 |

# **Supplementary Table S2**. ICD-10 Codes used to identify liver-related conditions and liver decompensation

| Liver Decompensation Variable | ICD-10 Code(s) |
| --- | --- |
| Alcoholic cirrhosis | K70.3 |
| Cirrhosis, other & unspecified | K74.6 |
| GI hemorrhage | K92.0, K92.1, K92.2, K62.5, I85.0, I85.9, I86.4, K29.0, K29.2, K29.5, K29.7, K29.8, K20, K21.0, K25.0, K25.3, K25.4, K25.7, K25.9, K26.4, K26.9, K27.9, K22.6, K22.1 |
| HRS/renal failure | K76.7, N17.0, N17.8, N17.9 |
| Ascites (excluding AH or cirrhosis in (1) and (2)) | R18 |
| Hepatic encephalopathy | K72 |
| Alcoholic hepatic failure | K70.4 |
| Alcohol Use Disorder (AUD) | F10.1, F10.2 |
| Alcohol abuse, dependence or withdrawal | F10.1, F10.2, F10.3 |

**Supplementary Table S3**. Factors Associated with Time to Liver Transplantation within 3 Years Among Patients Who Received Liver Transplantation

| Variable | Adjusted Hazard Ratio (HR) | Lower 95 Confidence Limit | Upper 95% Confidence Limit |
| --- | --- | --- | --- |
| Female | 1.0551 | 0.9782 | 1.1382 |
| Age | 0.9901 | 0.9870 | 0.9933 |
| Race/Ethnicity (ref: White) |  |  |  |
| Hispanic ethnicity | 1.0689 | 0.9371 | 1.2193 |
| African American race | 1.0418 | 0.8801 | 1.2333 |
| Asian race | 0.9609 | 0.7583 | 1.2178 |
| Unknown race | 1.0269 | 0.9389 | 1.1233 |
| Diabetes (ref: No diabetes) |  |  |  |
| Diabetes | 0.7337 | 0.6628 | 0.8122 |
| Diabetes with complications | 0.6883 | 0.5874 | 0.8065 |
| Cancer (ref: No cancer) |  |  |  |
| Non-metastatic liver cancer | 1.9891 | 1.7616 | 2.2460 |
| Metastatic liver cancer | 1.6679 | 0.9992 | 2.7841 |
| Other non-metastatic cancer | 0.7627 | 0.5507 | 1.0564 |
| Other metastatic cancer | 1.9646 | 0.9790 | 3.9426 |
| Myocardial infarction | 1.4461 | 0.9912 | 2.1099 |
| Congestive heart failure | 0.9504 | 0.7849 | 1.1507 |
| Peripheral vascular disease | 0.9364 | 0.7714 | 1.1367 |
| Cerebral vascular disease | 0.9612 | 0.6957 | 1.3280 |
| Dementia | 2.5506 | 1.0492 | 6.2003 |
| COPD | 1.0014 | 0.8587 | 1.1679 |
| Rheumatoid disease | 0.5529 | 0.4009 | 0.7625 |
| Peptic ulcer disease | 1.3135 | 1.0318 | 1.6721 |
| Hemiplegia | 1.4199 | 0.5828 | 3.4592 |
| HIV/AIDS | 0.7798 | 0.4380 | 1.3882 |
| Renal disease | 1.2604 | 1.0979 | 1.4469 |
| Alcoholic-associated cirrhosis | 0.7065 | 0.6251 | 0.7984 |
| Other & unspecified cirrhosis | 0.5386 | 0.4776 | 0.6074 |
| GI hemorrhage | 0.8535 | 0.7753 | 0.9396 |
| Hepatorenal syndrome | 1.9783 | 1.7275 | 2.2655 |
| Ascites | 1.7316 | 1.5907 | 1.8850 |
| Hepatic Encephalopathy | 1.6899 | 1.5391 | 1.8555 |
| Alcoholic Hepatic Failure | 1.1467 | 0.9074 | 1.4492 |
| Alcohol use disorder | 1.0507 | 0.9338 | 1.1822 |
